# A “Dirty” Footprint: Macroinvertebrate diversity in Amazonian Anthropic Soils

**DOI:** 10.1111/gcb.15752

**Published:** 2021-07-10

**Authors:** Wilian C. Demetrio, Ana C. Conrado, Agno N. S. Acioli, Alexandre C. Ferreira, Marie L. C. Bartz, Samuel W. James, Elodie da Silva, Lilianne S. Maia, Gilvan C. Martins, Rodrigo S. Macedo, David W. G. Stanton, Patrick Lavelle, Elena Velasquez, Anne Zangerlé, Rafaella Barbosa, Sandra C. Tapia‐Coral, Aleksander W. Muniz, Alessandra Santos, Talita Ferreira, Rodrigo F. Segalla, Thibaud Decaëns, Herlon S. Nadolny, Clara P. Peña‐Venegas, Cláudia M. B. F. Maia, Amarildo Pasini, André F. Mota, Paulo S. Taube Júnior, Telma A. C. Silva, Lilian Rebellato, Raimundo C. de Oliveira Júnior, Eduardo G. Neves, Helena P. Lima, Rodrigo M. Feitosa, Pablo Vidal Torrado, Doyle McKey, Charles R. Clement, Myrtle P. Shock, Wenceslau G. Teixeira, Antônio C. V. Motta, Vander F. Melo, Jeferson Dieckow, Marilice C. Garrastazu, Leda S. Chubatsu, TPI Network, Peter Kille, George G. Brown, Luís Cunha

**Affiliations:** ^1^ Department of Soil Science Federal University of Paraná Curitiba PR Brazil; ^2^ Biochemistry Department Federal University of Paraná Curitiba PR Brazil; ^3^ Federal University of Amazonas Manaus AM Brazil; ^4^ Entomology Department Federal University of Paraná Curitiba PR Brazil; ^5^ Centre for Functional Ecology Department of Life Sciences University of Coimbra Coimbra Portugal; ^6^ Maharishi International University Fairfield IA USA; ^7^ Embrapa Florestas Colombo PR Brazil; ^8^ Embrapa Amazônia Ocidental Manaus AM Brazil; ^9^ Instituto Nacional do Semiárido Campina Grande PB Brazil; ^10^ Department of Bioinformatics and Genetics Swedish Museum of Natural History Stockholm Sweden; ^11^ Institut de Recherche pour le Développement Cali Colombia; ^12^ Universidad Nacional de Colombia Palmira Colombia; ^13^ Ministère de l’Agriculture, de la Viticulture et de la Protection des consommateurs Luxembourg Luxembourg; ^14^ Centro Universitário do Norte Manaus AM Brazil; ^15^ Servicio Nacional de Aprendizaje SENA Regional Amazonas Leticia Colombia; ^16^ CEFE Univ Montpellier CNRS EPHE IRD Univ Paul‐Valéry Montpellier Montpellier France; ^17^ Instituto Amazónico de Investigaciones Científicas SINCHI Leticia Colombia; ^18^ Universidade Estadual de Londrina Londrina PR Brazil; ^19^ Universidade Federal do Oeste do Pará Pará Brazil; ^20^ Instituto Nacional de Pesquisas da Amazônia Manaus AM Brazil; ^21^ Embrapa Amazônia Oriental Santarém PA Brazil; ^22^ Museu de Arqueologia e Etnologia Universidade de São Paulo São Paulo SP Brazil; ^23^ Museu Paraense Emílio Goeldi Belém PA Brazil; ^24^ Soil Science Department Escola Superior de Agricultura Luís de Queiroz Universidade de São Paulo Piracicaba SP Brazil; ^25^ Embrapa Solos Rio de Janeiro RJ Brazil; ^26^ School of Biosciences Cardiff University Cardiff CF UK; ^27^ School of Applied Sciences University of South Wales Pontypridd CF UK; ^28^ Present address: INPE – National Institute for Space Research São José dos Campos SP 12227‐010 Brazil

**Keywords:** Amazonian Dark Earths, ants, archeological sites, disturbance, earthworms, land‐use change, soil fauna, soil fertility, termites, Terra Preta

## Abstract

Amazonian rainforests, once thought to be pristine wilderness, are increasingly known to have been widely inhabited, modified, and managed prior to European arrival, by human populations with diverse cultural backgrounds. Amazonian Dark Earths (ADEs) are fertile soils found throughout the Amazon Basin, created by pre‐Columbian societies with sedentary habits. Much is known about the chemistry of these soils, yet their zoology has been neglected. Hence, we characterized soil fertility, macroinvertebrate communities, and their activity at nine archeological sites in three Amazonian regions in ADEs and adjacent reference soils under native forest (young and old) and agricultural systems. We found 673 morphospecies and, despite similar richness in ADEs (385 spp.) and reference soils (399 spp.), we identified a tenacious pre‐Columbian footprint, with 49% of morphospecies found exclusively in ADEs. Termite and total macroinvertebrate abundance were higher in reference soils, while soil fertility and macroinvertebrate activity were higher in the ADEs, and associated with larger earthworm quantities and biomass. We show that ADE habitats have a unique pool of species, but that modern land use of ADEs decreases their populations, diversity, and contributions to soil functioning. These findings support the idea that humans created and sustained high‐fertility ecosystems that persist today, altering biodiversity patterns in Amazonia.

## Graphical abstract

Amazonian Dark Earths (ADEs) are fertile soils found throughout the Amazon Basin, created by pre‐Columbian societies with sedentary habits. Much is known about the chemistry of these soils, yet their zoology has been neglected. Studying nine archeological sites in Amazonian regions and adjacent reference soils we found 673 morphospecies and, we identified a tenacious pre‐Columbian footprint, with 49% of morphospecies found exclusively in ADEs. These findings support the idea that humans created and sustained high‐fertility ecosystems that persist today, altering biodiversity patterns in Amazonia.

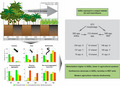

## 1 INTRODUCTION

The Amazon basin still contains the largest continuous and relatively well‐preserved tract of tropical forest on the planet. However, deforestation rates have been increasing over the last decade, resulting in the loss of an estimated 11.088 km^2^ of natural vegetation in 2020 alone (INPE, 2021). Many forested areas have become highly fragmented and may be reaching tipping points where biodiversity and ecosystem functions may be dramatically affected (Barkhordarian et al., 2018; Decaëns et al., 2018), potentially leading to cascading effects that impact ecosystem functioning over a much larger area (Lathuillière et al., 2018; Lawrence & Vandecar, 2015).

**FIGURE 1:**
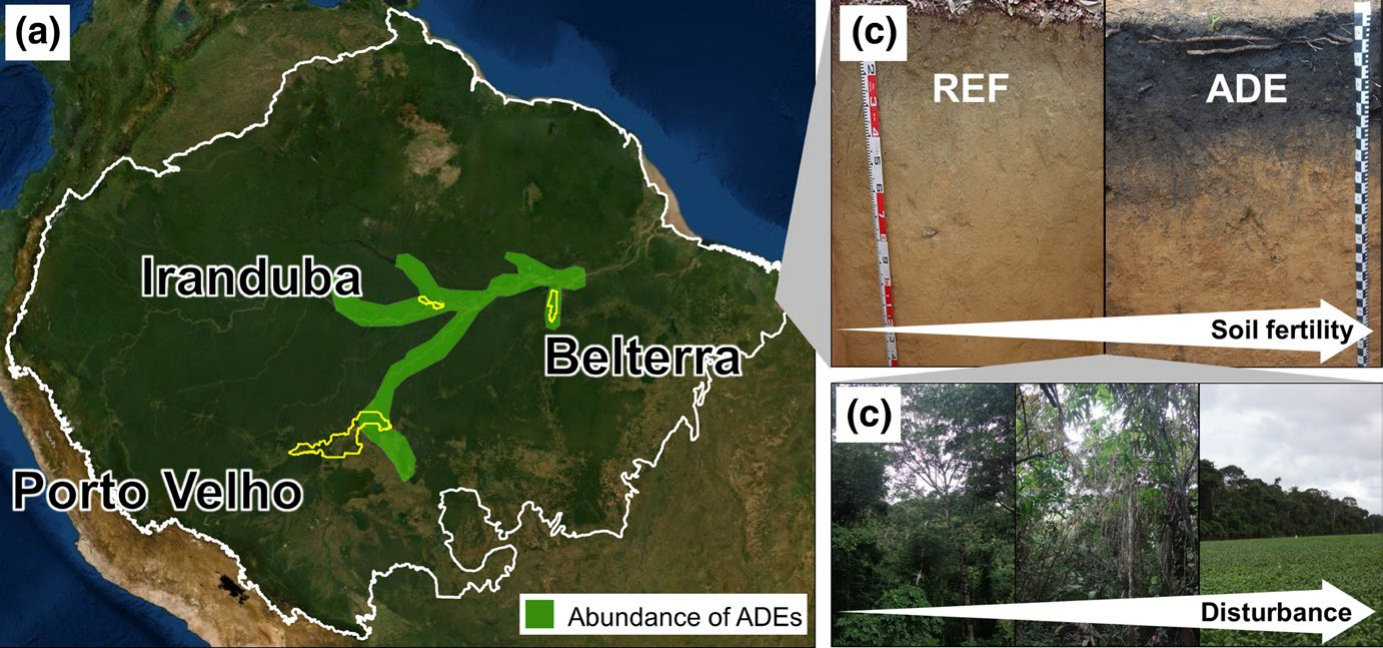
Sampling strategy to assess soil fauna and soil fertility in Central (Iranduba), Southwestern (Porto Velho), and Lower (Belterra) Amazon. (a) Boundary of Amazon Basin (white line), showing municipalities where samples were taken (boundaries in yellow lines), and areas with large occurrence of Amazonian Dark Earths (ADEs, shaded in green), modified from Clement et al. (2015). Amazonia map background: Esri, DigitalGlobe, GeoEye, Earthstar Geographics, CNES/Airbus DS, USDA, USGS, AEX, Getmapping, Aerogrid, IGN, IGP, swisstopo, and the GIS User Community. (b) Soil profiles of analytically paired ADE and nearby reference (REF) soils. The direction of the arrow shows the increase in soil fertility; Photos G.C. Martins, R. Macedo. (c) Land‐use systems sampled in each region, consisting in an intensification/disturbance gradient including older secondary rainforest (>20 years, undisturbed), young regeneration forest (<20 years old), and recent agricultural systems (pasture, soybean, and maize). The direction of the arrow shows the increase in contemporary anthropogenic disturbance. Photos G.C. Martins, M. Bartz

But humans have been modifying Amazonian biodiversity patterns over millennia. Native Amazonians created areas with high concentrations of useful trees and hyperdominance of some species, often associated with archeological sites (Levis et al., 2017, 2018; Ter Steege et al., 2013). Furthermore, occupations of some indigenous societies, beginning at least 6500 years ago, created fertile soils, locally called Amazonian Dark Earths (ADEs) or “*Terra Preta de Índio*” in Portuguese (Clement et al., 2015; Glaser, 2007; Glaser & Birk, 2012; McMichael et al., 2014; Watling et al., 2018; Figure 1b) that may occupy from 0.1 (Sombroek et al., 2003) up to 3% (McMichael et al., 2014) of the surface area of Amazonia. They appear to be more common along major rivers (Figure 1a) but are also abundant in interfluvial areas (Clement et al., 2015; Levis et al., 2020). ADE sites tend to have high contents of soil P, Ca, and pyrogenic‐C (Glaser & Birk, 2012; Lima et al., 2002; Sombroek et al., 2003), and host particular communities of plants and soil microorganisms (Brossi et al., 2014; Taketani et al., 2013). However, up to now soil animal communities in these anthropic soils are practically unknown, having been the target of only three studies of limited geographic scope (all sites near Manaus), focusing on earthworms (Cunha et al., 2016) and soil arthropods (Sales et al., 2007; Soares et al., 2011).

Soil macroinvertebrates represent as much as 25% of overall known described species (Decaëns et al., 2006), and may easily surpass 1 million species worldwide (Brown et al., 2018). However, soil animal communities have been little studied in megadiverse regions such as the Amazonian rainforest (Barros et al., 2006; Franco et al., 2018; Marichal et al., 2014), and these habitats may be home to thousands of described and still undescribed species (Brown et al., 2006), particularly smaller invertebrates such as nematodes and mites (Franklin & Morais, 2006; Huang & Cares, 2006) but also macroinvertebrates (Mathieu, 2004). Furthermore, these invertebrates may be particularly susceptible to land‐use changes such as deforestation (Decaëns et al., 2018; Franco et al., 2018; Mathieu et al., 2005) and can be used as bioindicators of both soil quality and of environmental disturbance (Gerlach et al., 2013; Lawton et al., 1998; Rousseau et al., 2013; Velásquez & Lavelle, 2019).

Hence, the aim of this study was to assess soil invertebrate macrofauna communities and their activity in nine ADEs classified as Anthrosols and nine non‐anthropic reference Amazonian Acrisols, Ferralsols and Plinthosols (referred to in this paper as REF soils) under three land‐use systems (LUS: old and young secondary forest and recent agricultural/pastoral systems; Figure 1c), to evaluate anthropic effects on soil biodiversity. We predicted that (1) soil biodiversity and soil enrichment in anthropic soils would reflect a unique habitat (explained by a pre‐Columbian footprint) but also that (2) animal species richness, biomass, and activity, as well as nutrient contents in these soils, would be determined by present‐day land use.

## 2 MATERIALS AND METHODS

### 2.1 Study sites

Our study was performed in three regions (central, lower, and southwestern Amazonia) of Brazilian Amazonia, with sampling conducted in Iranduba county in central Amazonia, Belterra county in lower Amazonia, and Porto Velho in southwestern Amazonia (Figure 1a; Table 1). All regions have a tropical monsoon (Köppen's Am) or without dry season (Köppen's Af) climate, with a mean annual temperature of 24–26.7℃ and precipitation between 2000 and 2420 mm year^−1^ (Alvares et al., 2013). In each region, paired sites with ADEs and nearby non‐anthropic REF soils (Figure 1b) were selected under different land‐use systems (Figure 1c): native secondary vegetation (*dense ombrophilous forest*) classified as old secondary forest when >20 years old, or young regeneration forest when <20 years old, and agricultural systems of maize in Iranduba, soybean in Belterra, and introduced pasture in Porto Velho. The REF sites were located within a minimum distance of 150 m (soybean at Belterra) to a maximum distance of 1.3 km (pasture at Porto Velho) from the ADE sites, and maximum distance between the three sampling locations within a region was 14 km (Embrapa sites to Tapajós National Forest sites in Belterra), totaling 18 sampled sites (3 regions × 3 land‐use systems × 2 soil types).

**TABLE 1:** Land‐use system, age of modern human intervention, soil type, and soil category according to IUSS (2015) and location of the sites studied in three regions of Brazilian Amazonia

Region	State	Land use	Human intervention^a^	Soil type	Soil category	Coordinates
Iranduba	AM	Old forest	>20 years old	REF	Xanthic Dystric Acrisol	3°14′49.00″S, 60°13′30.71″W
		Old forest	>20 years old	ADE	Pretic Clayic Anthrosol	3°15′11.05″S, 60°13′45.03″W
		Young forest	<20 years old	REF	Xanthic Dystric Acrisol	3°13′34.47″S, 60°16′23.60″W
		Young forest	<20 years old	ADE	Pretic Clayic Anthrosol	3°13′49.23″S, 60°16′7.43″W
		Agricultural	Current	REF	Xanthic Dystric Acrisol	3°13′31.31″S, 60°16′29.18″W
		Agricultural	Current	ADE	Pretic Clayic Anthrosol	3°13′46.13″S, 60°16′7.32″W
Belterra	PA	Old forest	>20 years old	REF	Xanthic Dystric Ferralsol	2°47′4.59″S, 54°59′53.28″W
		Old forest	>20 years old	ADE	Pretic Clayic Anthrosol	2°47′3.25″S, 54°59′59.77″W
		Old forest	>20 years old	REF	Xanthic Dystric Acrisol	2°41′13.90″S, 54°55′3.30″W
		Old forest	>20 years old	ADE	Pretic Clayic Anthrosol	2°41′7.18″S, 54°55′7.11″W
		Agricultural	Current	REF	Xanthic Dystric Acrisol	2°41′3.56″S, 54°55′12.75″W
		Agricultural	Current	ADE	Pretic Clayic Anthrosol	2°41′3.79″S, 54°55′7.90″W
Porto Velho	RO	Young forest	<20 years old	REF	Xanthic Dystric Plinthosol	8°52′11.50″S, 64°03′18.16″W
		Young forest	<20 years old	ADE	Pretic Clayic Anthrosol	8°51′51.92″S, 64°03′48.03″W
		Young forest	<20 years old	REF	Xanthic Dystric Ferralsol	8°50′49.52″S, 64°03′59.20″W
		Young forest	<20 years old	ADE	Pretic Clayic Anthrosol	8°51′1.18″S, 64°04′3.07″W
		Agricultural	Current	REF	Xanthic Dystric Ferralsol	8°52′35.30″S, 64°03′58.58″W
		Agricultural	Current	ADE	Pretic Clayic Anthrosol	8°51′56.53″S, 64°03′40.67″W

Abbreviations: ADE, Amazonian Dark Earth; REF, reference soil.

^a^ Age of modern human disturbance (land management).

One of the two old secondary forest sites in Belterra was at the Embrapa Amazônia Oriental Belterra Experiment Station, whereas the other one was at the Tapajós National Forest, a site of previous work on ADEs (Maezumi et al., 2018). The old secondary forests (ADE and REF) in Iranduba were at the Caldeirão Experimental Station of Embrapa Amazônia Ocidental and have been extensively studied in the past for soil fertility and pedogenesis (Alho et al., 2019; Macedo et al., 2017), as well as for soil microbial diversity (Germano et al., 2012; Grossman et al., 2010; Lima et al., 2014, 2015; O’Neill et al., 2009; Taketani et al., 2013). Initial and partial results of the earthworm data from the young and old forests, and the maize fields in Iranduba, were presented in an earlier publication (Cunha et al., 2016). ADE formation in Iranduba was estimated to have begun ~1050–950 years bp (Macedo, 2014; Neves et al., 2004) and at Belterra ~530–450 years bp (Maezumi et al., 2018). At Porto Velho, ADE formation began much earlier (~6500 years bp; Watling et al., 2018).

The agricultural fields with annual crops were under continuous (at least 6 years) annual row cropping of maize (Iranduba) and soybean (Belterra) and had been planted <60 days prior to sampling, using conventional tillage (Iranduba) or reduced tillage (Belterra). The crops received the recommended doses of inorganic fertilizers and pesticides for each crop; all crops were planted using certified commercial seeds. The pastures at Porto Velho were around 9 year old (REF) and 12 year old (ADE) and planted with *Brachiaria* (REF) and *Paspalum* (ADE) grasses. Soils at most REF sites were classified according to the World Reference Base for Soil Resources—WRB (IUSS, 2015) as dystrophic Ferralsols and Acrisols (Table 1), the two most common soil types in Amazonia (Gardi et al., 2015). At one young regeneration forest site in Porto Velho, both ADE and REF soil horizons were overlying a plinthic horizon and the REF soil was classified as a Plinthosol. All ADEs were classified as Pretic Clayic Anthrosols, with dark organic‐matter‐rich surface soil horizons, generally >20 cm thick. All soils had greater than 50% clay and clayey texture.

### 2.2 Soil macroinvertebrate sampling

We performed field sampling in April (Iranduba) and May (Belterra) of 2015, and in late February and early March of 2016 (Porto Velho), at the end of the main rainy season, which is the best time to collect soil macroinvertebrates (Swift & Bignell, 2001). Soil and litter macrofauna were collected using the ISO (2011) standard method devised by the Tropical Soil Biology and Fertility (TSBF) Program of the United Nations Educational, Scientific and Cultural Organization (UNESCO; Anderson & Ingram, 1993), and considered appropriate for evaluating soil macrofauna populations in the tropics. At each one of the 18 sampling sites, five sampling points were located within a 1 ha plot, at the corners and the center of a 60 × 60 m square, resulting in an “X”‐shaped sampling design (Figure S1). At each of these points, a soil monolith (25 × 25 cm up to 30 cm depth) was initially delimited with a 10 cm deep steel template, and then divided into surface litter and three 10 cm thick soil layers (0–10, 10–20, and 20–30 cm), totaling 90 soil monoliths that generated 270 soil layers + 90 litter samples. Macroinvertebrates (i.e., invertebrates visible to the naked eye and with generally >2 mm body width) were collected in the field by hand‐sorting both the soil and surface litter and were immediately fixed in 92% ethanol. Earthworms, ants, and termites were identified to species or morphologically different morphospecies (generally with genus‐level assignations) by co‐authors SWJ and MLCB (earthworms), ACF and RMF (ants), and ANSA (termites), while the remaining macroinvertebrates were sorted into morphospecies within higher taxonomic level assignations (e.g., order and/or family).

### 2.3 Additional samples for ecosystem engineers

As ecosystem engineers (earthworms, termites, and ants) represent most of the soil macrofauna collected in Amazonian soils (Barros et al., 2006), and we expected them to also be important at the study sites, we performed additional sampling for earthworms, termites, and ants, in order to better estimate their species richness, especially in forest sites, where higher diversity was expected. In all sampling sites, extra earthworm samples were collected at four additional cardinal points of the grid (Figure S1), by hand‐sorting soil from holes of similar dimensions as the TSBF monoliths and the individuals collected preserved in 96% ethanol. The extra termite samples were collected in old secondary forests and young regeneration forests (except at one of the REF young forests in Porto Velho), but not in the agricultural fields (maize, soybean, and pasture) where there are very few termite colonies and the TSBF monoliths would capture most of the species present. Termites were sampled in five 20 m^2^ (2 × 10 m) plots near the TSBF monoliths (Figure S1) by manually digging the soil and looking for termitaria in the soil, as well as in the litter and on trees using a modification of the transect method of Jones and Eggleton (2000), totaling 50 extra samples from 10 sites. Extra samples for ants were taken only in the forest systems of Iranduba and Belterra. Ants were sampled in 10 pitfall traps (300 ml plastic cups) set up as two 5‐trap transects on the sides of each 1 ha plot, with 20 m distance between traps (Figure S1), as well as in two traps to the side of each TSBF monolith (distant ~5 m), totaling 20 pitfall traps in each site and 160 samples in total. Each cup was filled to a third of its volume with water, salt, and detergent solution. The pitfall traps remained in the field for 48 h. Termites and ants were preserved in 80% ethanol and the alcohol changed after cleaning the samples within 24 h. All the animals (earthworms, ants, and termites) were identified to species level or morphospecies level by co‐authors as described above.

### 2.4 Soil physical and chemical attributes

After hand‐sorting the soil fauna from each TSBF monolith, 2‐ to 3‐kg soil samples were collected from each depth (0–10, 10–20, and 20–30 cm) for chemical and soil particle size analysis, and although analyzed separately, mean values were calculated over 0–30 cm depth. The following soil properties were assessed using standard methodologies (Teixeira et al., 2017): pH (CaCl_2_); Ca^2+^, Mg^2+^, and Al^3+^ (KCl 1 mol L^−1^); K^+^ and available P (Mehlich‐1); total nitrogen (TN) and carbon (TC) by combustion (CNHS). Base saturation, sum of bases (SB) and cation exchange capacity (CEC) were calculated using standard formulae (Teixeira et al., 2017). Soil texture was determined according to the FAO soil texture triangle and the particle size fractions (% sand, silt, and clay) obtained following standard methodologies (Teixeira et al., 2017).

To assess functional differences induced by soil fauna activity in the ADE and REF soils, soil macromorphology samples were taken 2 m away from each monolith (Figure S1) using a 10 × 10 × 10 cm metal frame. The collected material was separated into different fractions including living invertebrates, litter, roots, pebbles, pottery sherds, charcoal (biochar) fragments, non‐aggregated/loose soil, physical aggregates, root‐associated aggregates, and fauna‐produced aggregates (generally with rounded shapes and darker color than other aggregates) using the method of Velásquez et al. (2007). Each fraction was oven‐dried at 60℃ for 24 h and weighed. This method allows estimating the relative contribution of soil macrofauna, roots and soil physical processes to soil macroaggregation and structure, that determine the delivery of important soil‐based ecosystem services such as carbon sequestration, water infiltration and availability in the soil, and erosion and flood control (Adhikari & Hartemink, 2016; Velásquez & Lavelle, 2019).

### 2.5 Treatment of soil fauna data

Density (number of individuals) and biomass of the soil macrofauna surveyed using the TSBF method were extrapolated per square meter considering all depths evaluated. Density and biomass of immature forms of insects (nymphs and larvae) were grouped in the respective taxonomic group. The following taxonomic groups, representing 2% or less of total density, were grouped as “Others”: Araneae, Hemiptera, Orthoptera, Diptera (larvae), Gastropoda, Dermaptera, Isopoda, Blattaria, Scorpiones, Opiliones, Lepidoptera (larvae), Thelyphonida, Solifugae, Thysanoptera, Geoplanidae, Neuroptera (larvae), Hirudinea, Diplura, Vespidae, and Embioptera. The earthworms, ants, and termites were also combined into the category of ecosystem engineers (Jones et al., 1994; Lavelle et al., 1997). To calculate the beta (*β*) diversity index, we removed singleton species (species represented by single individuals, i.e., one individual among all the 8378 individuals collected).

### 2.6 Statistical analyses

To compare species richness between ADE and REF, we plotted rarefaction and extrapolation curves based on the Chao1 index (Chao, 1984) using the iNEXT package (Hsieh et al., 2016) for total macroinvertebrate, ant, termite, and earthworm morphospecies diversity, using the number of TSBF monolith samples as a measure of sampling effort intensity. The same procedure was used for all earthworm data (9 samples per site), termite data obtained from both the 20 m^2^ plots and TSBF monoliths, and ant data obtained from both pitfall traps and TSBF monoliths. Confidence intervals for rarefaction and extrapolation curves were obtained by running a bootstrapping procedure (999 iterations).

We used the betapart package (Baselga & Orme, 2012) in R (R Core Team, 2020) to decompose β‐diversity (calculated using the Sørensen dissimilarity index) into its Turnover (Simpson index of dissimilarity) and Nestedness components using the species presence/absence (binary data) of all soil and litter macroinvertebrate, ant, termite, and earthworm data from monolith samples. The average β‐diversity was calculated to highlight land‐use effects, by comparing all land‐use systems (old forests, young forests, and agriculture) within each soil type (REF and ADE) and region, thus isolating the land‐use effect. The soil type effect was assessed by comparing the diversity between REF and ADE soils within each land‐use system in each region. To identify the effect of geographic distance (region effect) on species turnover, we also calculated the average β‐diversity among the three replicates of each land‐use system within each soil type.

Due to non‐normal distribution of both the faunal variables (i.e., density and biomass of invertebrates collected using the TSBF method) and soil properties, we used Generalized Linear Models (GLiM) to adjust data to other probability distributions. The best adjustment was Poisson for invertebrate density and Gamma for invertebrate density biomass. Soil chemical properties and particle size fractions data were adjusted in Gamma. ANOVA tests were performed with the multcomp package (Hothorn et al., 2008) in R, adopting a factorial design with the following factors: soil type (ADE and REF) and LUS (old forests, young forests and agricultural systems). When factor interactions were significant (*p* < 0.05), the data were analyzed comparing the effects of soil type within the LUS and the effects of LUS within each soil type. Significant differences were tested using Tukey's test at 95% probability (*p* < 0.05).

A Non‐Metric Multidimensional Scaling (NMDS) analysis was performed in R (Oksanen et al., 2019) using the densities of earthworms, termites, ants (data from 0 to 10 cm layer) and overall morphospecies richness of ecosystem engineers (litter+0–30 cm depth), together with the results of five variables from soil macromorphology (non‐aggregated soil, pottery sherds, and fauna‐produced, root‐associated, and physical aggregates) and 6 variables from soil chemical analyses (pH, Al^3+^, P, SB, TC, and TN).

## 3 RESULTS

### 3.1 ADEs are distinct ecosystems

**FIGURE 2:**
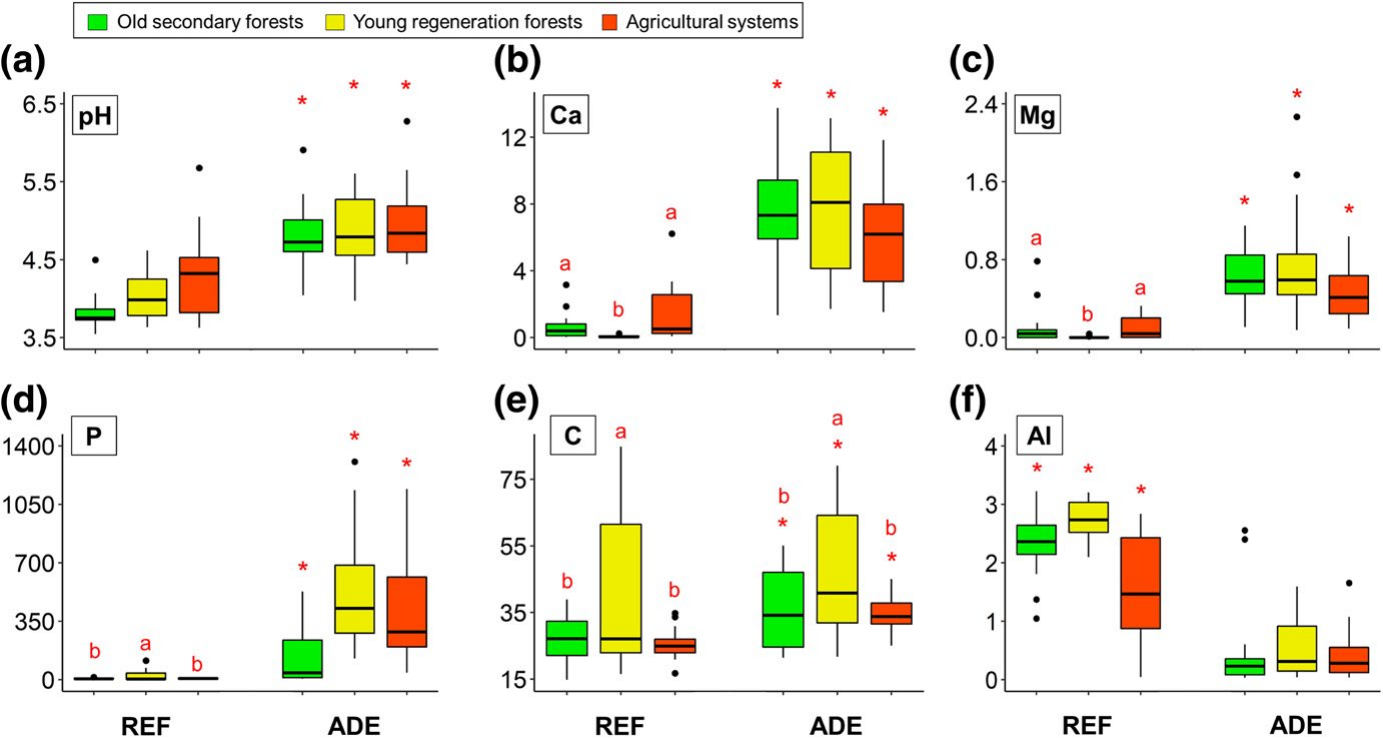
Soil chemical properties in the topsoil layer (0–30 cm depth; mean values for the three regions) at the collection sites in Amazonia: (a) pH (in Ca Cl_2_), (c) exchangeable Ca (cmol_c_ kg^−1^), (c) exchangeable Mg (cmol_c_ kg^−1^), (d) available P (mg kg^−1^), (e) total carbon (g kg^−1^), and (f) exchangeable Al (cmol_c_ kg^−1^) in each soil type (REF vs. ADE soils) and land‐use system (Secondary forests, Regeneration forests, Agricultural systems). Red asterisks indicate significant differences (*p* < 0.05) between soil categories (ADE vs. REF) within each land‐use system, while different lower‐case red letters indicate significant differences among land‐use systems within the same soil type. ADE, Amazonian Dark Earth; REF, reference soils. Values shown are median (black line), 1st and 3rd quartiles (box), max/min observations (upper and lower lines), and the outliers (small black circles), when present

The ADEs at all the sites had higher soil pH (Figure 2a) and were enriched in Ca, Mg, P, and total C compared to REF soils within each LUS (Figure 2b–e), following trends typically observed in ADE sites throughout Amazonia (Lehmann et al., 2003; Sombroek et al., 2003). Significantly lower amounts of exchangeable Al were also found in the ADEs (Figure 2f). Soil texture was similar in both ADE and REF soils from each site (Table S1), so the enrichment was not due to differences in clay contents, but was the result of ancient anthropogenic activities (Lehmann et al., 2003; Smith, 1980). Some differences in soil fertility among land‐use systems were also observed, where plots under annual cropping or pasture use in REF soils had higher Ca and Mg contents (due to liming) than the young regeneration forests (Figure 2b,c), as well as higher K contents and base saturation than in both young and old secondary forests (Table S1) due to fertilization. Total C and N contents were higher in young regeneration forests than in agricultural systems and old forests on both ADEs and REF soils (Figure 2e; Table S1), owing probably to high organic matter deposition in these rapidly regenerating young forests.

**FIGURE 3:**
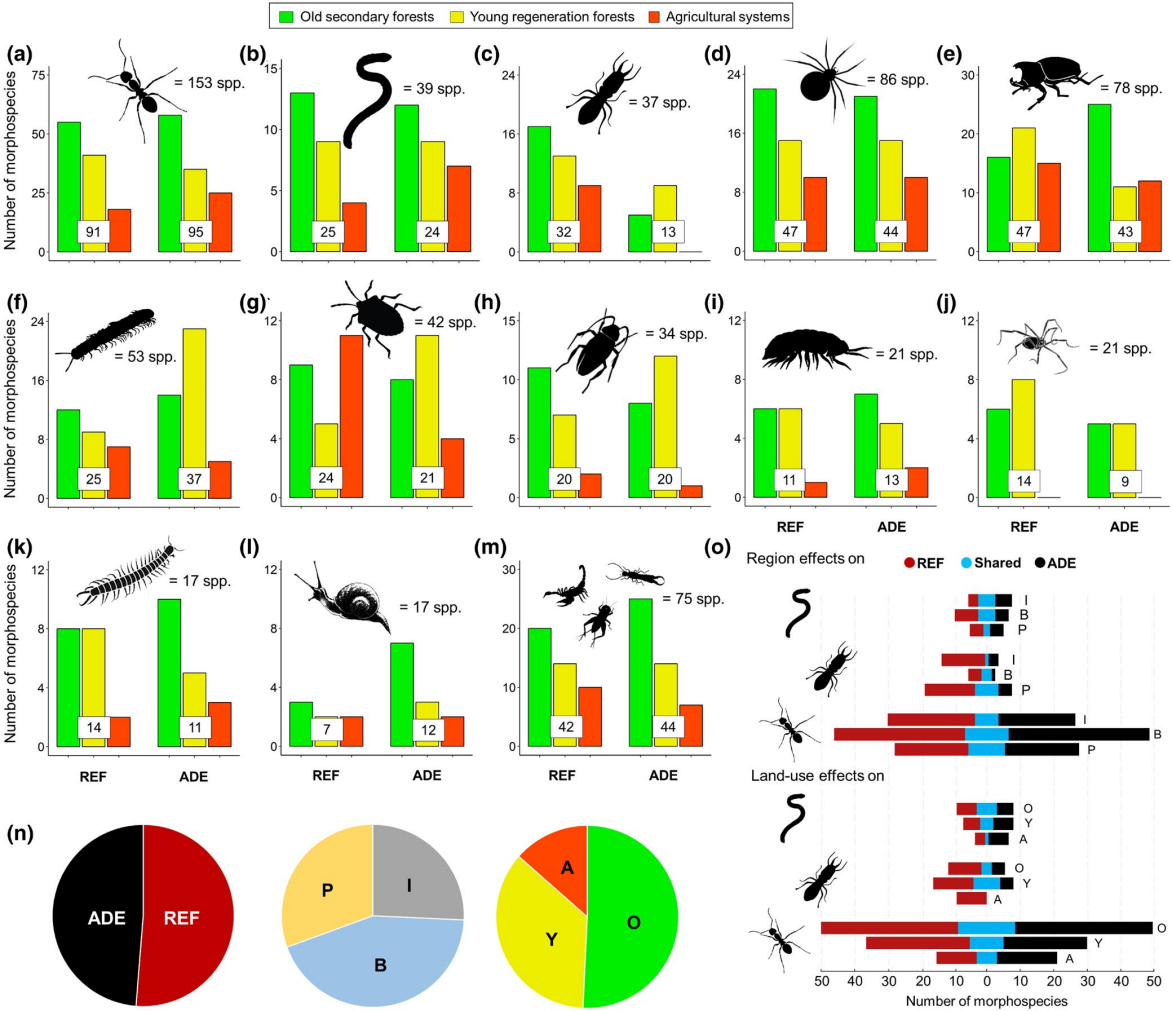
Morphospecies richness patterns in soil communities found in the monoliths dug at 18 collection sites in Amazonia: Total number of morphospecies of (a) ants, (b) earthworms, (c) termites, (d) spiders, (e) beetles (adults only), (f) millipedes, (g) true bugs, (h) cockroaches, (i) Isopods, (j) opilionids, (k) centipedes, (l) snails, and (m) others (sum of all remaining taxa encountered, including Dermaptera, Diplura, Diptera & Lepidoptera larvae, Embioptera, Geoplanidae, Hirudinea, Neuroptera, Orthoptera, Scorpiones, Solifugae, Thysanoptera, Thelyphonida, and Vespidae), according to soil type (ADE, REF) and land‐use systems. The total number of morphospecies of each taxon (a–m) found overall is shown on the top of each graph. (n) Distribution of morphospecies (including singletons) of all macroinvertebrates according to proportion (%) of unique species found in each soil type, region, and land‐use system. (o) Numbers of morphospecies of earthworms, termites, and ants observed in both soil categories (blue bars) or uniquely in ADE (black bars) or in REF (red bars) soils, in the different regions (I, B, P) and land‐use systems (O, Y, A) across regions. A, agricultural systems; ADE, Amazonian Dark Earth; B, Belterra; I, Iranduba; O, old secondary forests; P, Porto Velho; REF, reference soils; Y, young regeneration forests

We collected 8378 macroinvertebrates in soil monoliths, of 673 different morphospecies (Figure 3) belonging to 26 higher taxa. Ants were the most diverse group collected (153 spp.), followed by spiders (86 spp.), beetles (78 spp.), millipedes (53 spp.), true bugs (42 spp.), earthworms (39 spp.), termites (37 spp.), and cockroaches (34 spp.) (Figure 3, scientific names of higher taxa can be found in Demetrio et al., 2021). Less diverse taxa included isopods (21 spp.), opilionids (21 spp.), centipedes (17 spp.), and snails (17 ssp.) while the less abundant taxa (Others) represented a relatively species‐rich group, when combined (75 spp.). Furthermore, the number of singleton species (one individual in the total sample of 8378) was very high (336 spp.), representing 50% of total macroinvertebrate species richness (Table S2).

Species richness overall was similar in ADEs (385 spp.) and REF (399 spp.) soils, but more species were found in Belterra (314 spp., where two old forests were sampled) than in Porto Velho (238 spp., where both forests were young) and Iranduba (218 spp.). More than 50% of all morphospecies were present in old forests, compared with lower and much lower proportions, respectively, in young regeneration forests and agricultural systems (Figure 3n). From all the monoliths, total species richness of ants, earthworms, spiders, beetles, true bugs, cockroaches, and isopods was also fairly similar in each soil type (Figure 3a,b,d,e,g–i), but termite richness was much higher, and centipede and opilionid richness slightly higher, in REF than in ADE soils (Figure 3c,j,k). On the other hand, richness of both millipedes and snails was higher in ADE than REF soils (Figure 3f,l), possibly owing to the higher soil Ca levels found in ADEs (Figure 2b; Coleman et al., 2004).

The proportion of exclusive morphospecies was high in both soils: 49% in ADEs and 51% in REF soils (Figure 3n), particularly for ants (62 spp. were exclusive to ADE, 58 spp. exclusive to REF), spiders (39 spp. to ADE, 42 spp. to REF), beetles (31 spp. to ADE, 35 spp. to REF), true bugs (18 spp. to ADE, 21 spp. to REF), and earthworms (15 spp. to both ADE and to REF; Table S2; Figure 3o). Many more species of termites and opilionids were unique to REF soils (24 and 12 spp., respectively) than to ADE soils (5 and 7 spp., respectively), while many more species of millipedes and snails were unique to ADE soils (28 and 10 spp., respectively) than to REF soils (16 and 5 spp., respectively). These trends for ants, earthworms, and termites remained similar even after singleton species were removed (Table S2). Furthermore, among the ecosystem engineers collected, we found a considerable number of species new to science (>20 earthworm, >20 termite, and >30 ant species) that still must be formally described.

ADEs were home to 52 rare (which include doubletons and morphospecies with fewer than 10 ind. over all samples) and to 21 non‐rare or abundant macroinvertebrate morphospecies (taxa with ≥10 ind. over all samples) not found in REF soils (Table S2). Interestingly, within the non‐rare/abundant taxa, 16 species (of which seven were of ants and five were of earthworms) had greater abundance of individuals in ADEs, while 14 species (half of them ant species) were more abundant in REF soils (Table S2). Overall, very few species were shared between the paired ADE and REF soils at each site, with many species unique to each soil type (Figure S2).

**FIGURE 4:**
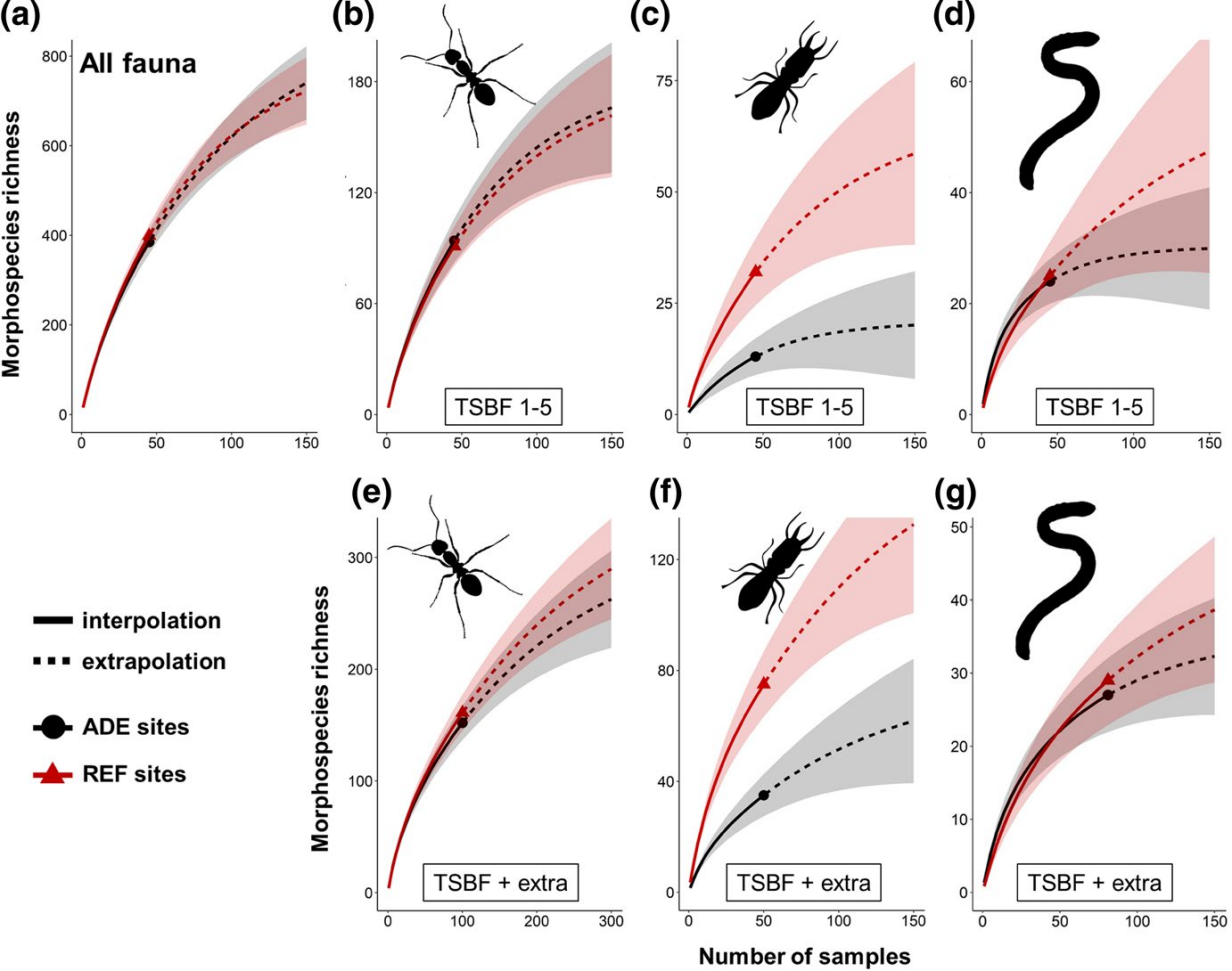
Morphospecies rarefaction and extrapolation curves, showing how morphospecies richness increases in both ADE and REF soils depending on sampling intensity (number of samples) for (a) all soil macroinvertebrates, (b) ants, (c) termites, and (d) earthworms considering only soil monolith (TSBF) samples; and for (e) ants collected in pitfall traps + monoliths (TSBF) in old secondary and young regeneration forests in Iranduba and Belterra (Porto Velho data excluded), (f) termites in soil monolith samples + 10 m^2^ plots in old secondary and young regeneration forests (except one young forest in Porto Velho) and (g) earthworms from all monoliths (*n* = 9 per plot) samples over all sites. Rarefaction and extrapolation curves were obtained based in Chao1 index. Dark grey and red areas represent 95% confidence intervals (bootstrapping procedure). ADE, Amazonian Dark Earth; REF, Reference soil

Based on our results from the monolith samples (*n* = 45 for each soil type), estimated richness (i.e., that would have been obtained with increased sampling effort) for total macroinvertebrates, for ants, and for earthworms (Figure 4a,b,d, respectively) was not different between REF and ADE soils. For termites, however, estimated richness was three times higher in REF soils (20 vs. 58 spp.; Figure 4c), and predicted to be attained with 300 samples, that is, more than three times the present sampling effort (90 samples). These results were confirmed with the additional samples taken for ants, termites, and earthworms, which showed little difference between soil types in the increase in richness of ants and earthworms compared to the monoliths, but large differences for termites (Figure 4e–g, respectively). The monolith samples (*n* = 45 for each soil type) covered 63% of the estimated richness (up to 100 samples) of total soil macroinvertebrates and ants in both soil types (Figure S3). Termite richness was slightly better estimated by the monoliths in REF soils (~71%) than in ADEs (~62%), while earthworm richness was relatively well sampled with soil monoliths (especially in ADEs), which collected 72%–90% of the estimated species richness (up to 100 samples) in both soil types (Figure S3). Nonetheless, the use of complementary sampling methods greatly increased the richness of ants (57–70 additional spp.) and of termites (26–50 additional spp.), and slightly increased that of earthworms (3–4 additional spp.) collected in both soils, revealing a large species pool of these soil engineers not adequately evaluated using only the TSBF method (Figure 4e–g). Furthermore, increasing the current sampling effort could still greatly increase total termite richness, particularly in REF soils.

The high number of species unique to each soil was reflected in high β‐diversity values and species turnover, ranging from 66% to 87% for all of the soil macroinvertebrates, depending on the region, LUS and soil type (Table 2). Interestingly, land‐use effects on macroinvertebrate species turnover rates were slightly higher than those of soil type, indicating that species turnover was more affected by land‐use change than by soil type (Table 2). Similar results were observed for earthworms, with much higher turnover rates (0.85 and 0.65 within REF and ADEs, respectively) due to LUS than due to soil type, particularly in old secondary forests. Conversely, soil type had a greater impact than land use on termite species turnover, while for ants, the effect of soil type on species turnover was mainly observed in old secondary forests. The species turnover among regions was also very high, especially for overall macroinvertebrates (all taxa) and for earthworms in both soils, implying a high number of macroinvertebrate species (and earthworms) locally endemic to different parts of Amazonia (Table 2). For ants, species turnover was higher in both forest types than in the agricultural systems, implying that agricultural systems include a larger proportion of widespread species common to all three sampling regions.

**TABLE 2:** Effects of region, land‐use system (LUS), and soil type (REF and ADE) on β‐diversity (without singletons) and species turnover rates of total soil macrofauna (339 morphospecies), ant, termite, and earthworm assemblages. Richness values used for the calculations are from the soil monoliths (TSBF)

Partitioned effect	Max div. (β_Sorensen_)	Turnover (β_Simpson dis_.)	Max div. (β_Sorensen_)	Turnover (β_Simpson dis_.)	Max div. (β_Sorensen_)	Turnover (β_Simpson dis_.)	Max div. (β_Sorensen_)	Turnover (β_Simpson dis_.)
	All fauna		Ants		Termites		Earthworms	
Region effect^1^								
In REF	0.87	0.84	0.87	0.81	0.83	0.47	0.93	0.90
In ADE	0.86	0.82	0.84	0.79	0.82^a^	0.76^a^	0.84	0.84
LUS effect^2^								
In REF	0.85	0.79	0.90	0.82	0.83	0.67	0.90	0.85
In ADE	0.82	0.74	0.85	0.80	0.80	0.39	0.72	0.65
Soil effect^3^								
In O	0.74	0.70	0.86	0.85	0.85	0.79	0.43	0.31
In Y	0.68	0.66	0.82	0.80	0.76	0.68	0.68	0.68
In A	0.74	0.68	0.77	0.61	—	—	0.83	0.83

Abbreviations: A, agricultural systems; ADE, Amazonian Dark Earth; O, old secondary forests; REF, Reference soil; Y, young regeneration forests.

^a^ Calculated using only O and Y forest sites.

^1^ Region: Mean regional effect, presented for each soil type and calculated by averaging all turnovers for each LUS, tested between regions (e.g., old forest at Iranduba vs. old forests at Belterra on REF soil).

^2^ LUS: Mean effect of all differences in land‐use systems, presented for each soil type and within each region, and then averaged across all regions (e.g., both young forests compared with pasture at Porto Velho).

^3^ Soil: Mean effect of soil type in each land‐use system, compared within each region (e.g., old forest in Belterra on ADE compared with old forest in Belterra on REF soil) and then averaged over all regions.

### 3.2 Ecosystem engineers dominate the soil fauna communities

**FIGURE 5:**
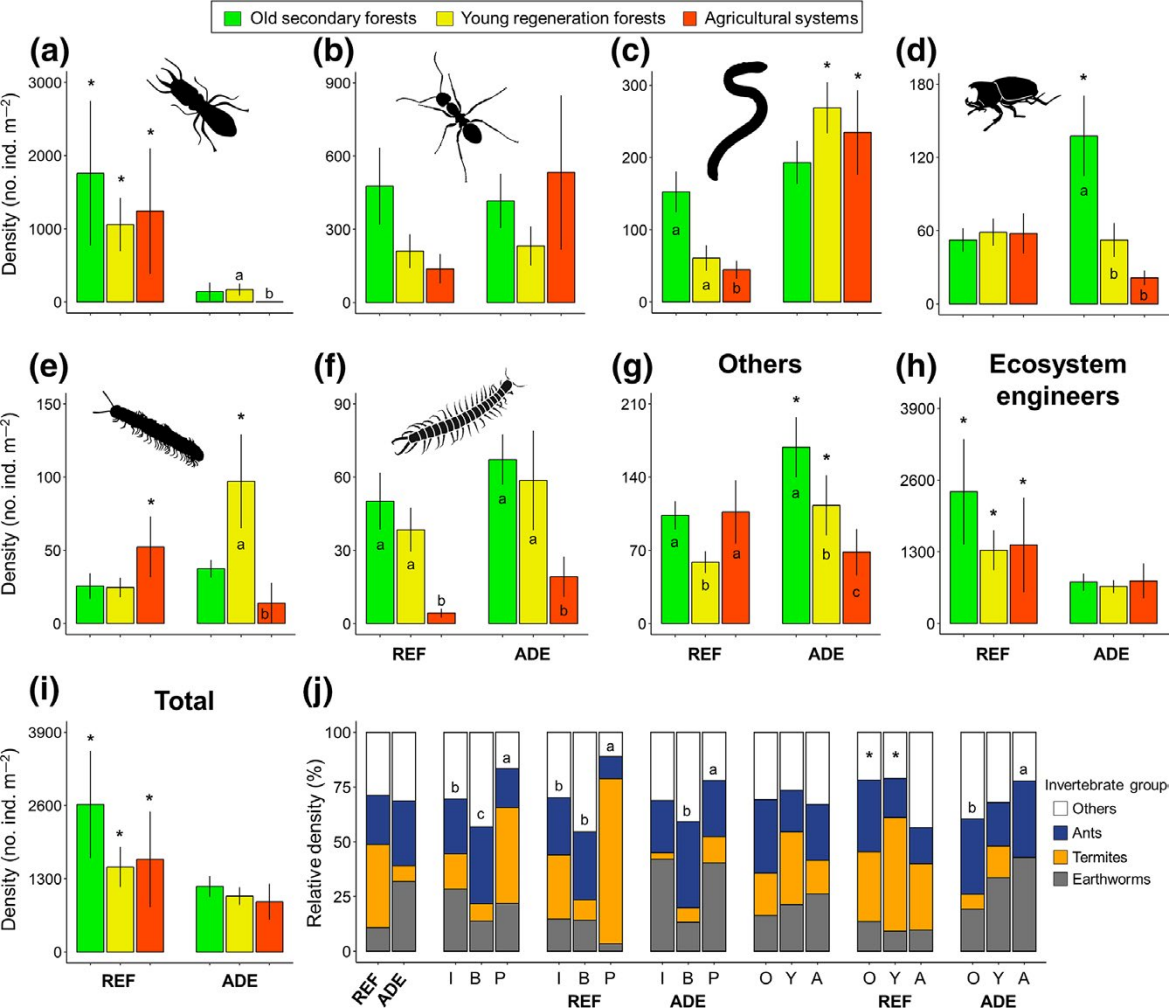
Mean density (Den.; number of individuals m^−2^) ± standard error of (a) termites, (b) ants, (c) earthworms, (d) beetles (adults + larvae), (e) millipedes, (f) centipedes, (g) others (all the remaining taxa), (h) Ecosystem engineers (i.e., earthworms, ants, and termites), and (i) total macroinvertebrates collected in each land‐use system studied, comparing REF and ADE soils. (j) Relative densities (%) of earthworms, termites, ants, and other soil macroinvertebrates (sum of all other taxa) found in the different soil categories (ADE and REF), regions (I, B, P), and land‐use systems (O, Y, A). Asterisks indicate significant differences (*p* < 0.05) in density between soils (ADE vs. REF) within each land‐use system, while different lower‐case letters indicate significant differences between land‐use systems within the same soil type, in the abundance of each taxonomic group (a–g). A, agricultural systems; ADE, Amazonian Dark Earth; B, Belterra; I, Iranduba; O, old secondary forests; P, Porto Velho; REF, reference soils; Y, young regeneration forests

Ecosystem engineers represented on average 72% and 69% of the soil macroinvertebrate individuals found in ADE and REF soils, respectively (Figure 5j). In the ADEs, earthworms represented from 13% to 43% of all individuals collected, while in the REF soils, termites represented 9% to 75% of total macroinvertebrate abundance, depending on the region and LUS. Ant proportions were less variable, ranging from 10% to 39% of total abundance. The proportion of ecosystem engineers was significantly higher in Porto Velho than in Iranduba and Belterra, mainly owing to the higher proportion of termites in Porto Velho (Figure 5j), particularly in REF soils. Earthworms were proportionately more abundant in Porto Velho (22%) and Iranduba (28%) than in Belterra, where the relative density of ants (35%) and non‐engineers (43% of total) was greater than in the two other regions.

The proportion of ecosystem engineer individuals found in each LUS was not different overall but varied in the ADE soil type, where there were proportionally more engineers in the agricultural systems than in the old forests (Figure 5j). Earthworms tended to be proportionally more important in ADEs while termites were more important in REF soils. Furthermore, engineers were significantly more abundant in REF than ADE soils of all land‐use systems (Figure 5h), mainly due to the termite populations that were significantly higher in REF soils of all LUS, with populations over 1000 individuals m^−2^ (Figure 5a). Meanwhile, with lower total populations, the earthworm abundance in both agricultural systems and young regeneration forests was significantly higher in ADEs than in REF soils (Figure 5c). Additionally, the abundance of beetles and other macroinvertebrates was higher in old forests on ADEs than REF soils and young forests or agricultural systems on ADEs (Figure 5d,g). Also, the abundance of millipedes was higher in young regeneration forests on ADEs than on REF soils (Figure 5e).

Ecosystem engineers represented from 65% to 94% of total soil fauna biomass, with earthworms being the largest component, representing 61%–99% of the engineer biomass and 44%–92% of the total macroinvertebrate biomass (Table S3). In both agricultural systems and in the young regeneration forests, earthworm biomass was higher on ADEs than on REF soils. Furthermore, in the young regeneration forests, ecosystem engineer, millipede, other and total macrofauna biomass were also significantly higher on ADEs than on REF soils (Table S3). On the other hand, in all LUS, termite biomass was significantly higher on REF soils than on ADEs. No other higher taxon of soil animals represented more than 16% of the total macroinvertebrate biomass in any given soil type or LUS (Table S3).

### 3.3 Soil biota influence ADE soil properties

**FIGURE 6:**
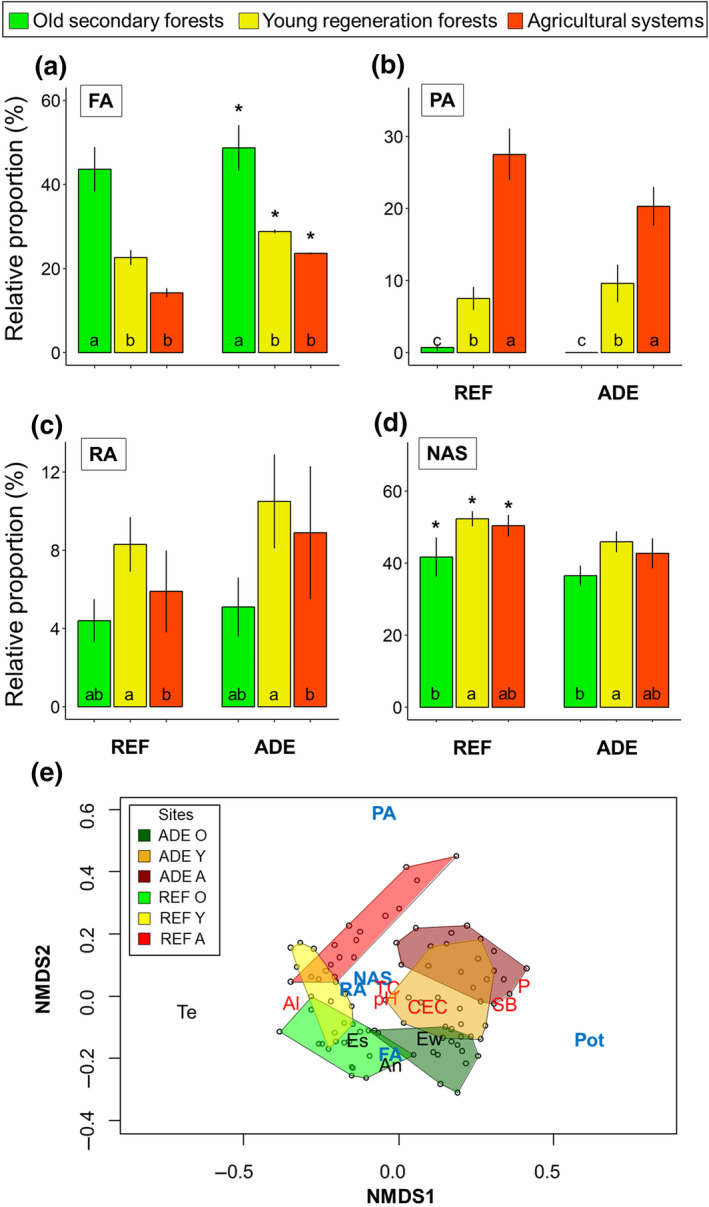
Macromorphological aggregate fractions (%) and their relationships to various soil attributes (0–10 cm layer) in two different Amazonian soils (ADE, Amazonian Dark Earth; REF, non‐anthropogenic reference soils) and three different land‐use systems (A, agricultural systems; O, old secondary forests; Y, young regeneration forests); (a) fauna‐produced aggregates (FA), (b) physical aggregates (PA), (c) root aggregates (RA), (d) non‐macroaggregated loose soil particles and unidentified aggregates less than 5 mm in size (NAS). Values shown are relative mean (%) ± standard error. Asterisks indicate significant differences (*p* < 0.05) between soil categories within each land‐use system, while different lower‐case letters indicate significant differences between land‐use systems within the same soil type. (e) Non‐metric Multidimensional Scaling (NMDS) of soil macroinvertebrate data, combined with soil macromorphology features and soil chemical properties: Blue letters: macromorphological fractions (FA, fauna‐produced aggregates; NAS, non‐aggregated soil; PA, physical aggregates; Pot, Pottery sherds; RA, root‐associated aggregates). Black letters: density (no. ind. m^−2^) of ants (An), termites (Te) and earthworms (Ew), and overall ecosystem engineer morphospecies richness (Es). Red letters: soil chemical properties (Al, exchangeable aluminum; CEC, cation exchange capacity; P, available phosphorus, pH; SB, sum of bases; TC, total carbon)

Soil macromorphology revealed a significantly higher proportion of fauna‐produced aggregates in ADEs compared to REF soils (Figure 6a), and likewise, for samples from the same LUS, a lower proportion of non‐aggregated soil in ADEs than in REF soils (Figure 6d), implying important changes in soil structure in ADEs driven by soil macrofauna bioturbation.

The multivariate analysis (NMDS; Figure 6e) confirmed the importance of soil fertility variables (particularly Al, sum of bases, and available P contents) in separating ADE and REF soils, and the differences in macrofauna communities between the two (more earthworms in ADEs and more termites in REF soils). Furthermore, the analysis confirmed the role of land‐use disturbance or intensification (the LUS were aligned with the y‐axis) as a regulator of ecosystem engineer biodiversity and the types of aggregates present in the soil, with physical aggregates being more associated with agricultural systems and fauna‐produced aggregates with the more conserved forest ecosystems. Pottery sherds were found only in ADE soils, and these are relevant components of ADEs and in their classification (Kämpf et al., 2009).

### 3.4 Modern land use erodes soil biodiversity and function

Modern agricultural systems had lower richness of all major soil animal taxa (except for true bugs and snails in REF soils and beetles in ADEs; Figure 3) than both forest types (old and young), regardless of soil type (both ADE and REF). Total morphospecies richness at each site ranged from 51 (REF, Iranduba) to 91 (ADE, Belterra) in old secondary forests, from 37 to 80 in young regeneration forests (both ADE sites in Porto Velho) and from 18 (maize on ADE in Iranduba) to 44 (soybean on ADE in Belterra) in agricultural ecosystems (Figure S2). Over all sites 350, 278, and 151 morphospecies of macroinvertebrates were found in old and young forests and agricultural systems, respectively, of which 237, 167, and 63 species were unique to each respective LUS (Figure 3n). Removing singleton species, morphospecies richness was 135 (old forests), 97 (young forests), and 50 (agricultural systems) in ADE soils, and 119 (old forests), 96 (young forests), and 55 (agricultural systems) in REF soils. Hence, richness was 63% and 55% lower in modern agricultural systems compared with old and young forests, respectively. This trend was also observed for most of the groups of soil animals taken individually and was particularly marked (>60% decrease in species richness) for opilionids, centipedes, isopods, and cockroaches in both REF and ADE soils, and for earthworms in REF and termites in ADE soils (Figure 3). Species richness decreases in agricultural systems compared to old forests were slightly (but not significantly) higher for ADE (66%) than REF (56%) soils.

Abundance of predators (centipedes, arachnids, diplurans, earwigs, scorpions, opilionids, whip scorpions, solifuges, antlion larvae, leeches, and wasps) and of several individual taxa were also significantly lower in agricultural systems (Figure 5) compared with young forests (termite and millipedes on ADEs) and old forests (beetles, centipedes, and others on ADEs), or compared with both forest systems (earthworms and centipedes on REF soils), highlighting the negative impact of more intensive ecosystem disturbance on the populations of these taxa.

Furthermore, within each soil type, fauna‐produced aggregates were more abundant in the old forests compared to the young forests and agricultural systems (Figure 6a), which had significantly higher proportions of physical aggregates (Figure 6b). Root‐associated aggregates and non‐aggregated soil fractions were more abundant in young forests than agricultural systems (Figure 6c), and old forests (Figure 6d), respectively, implying important differences in soil structure dynamics in each LUS, with lower overall biotic contributions to soil functioning in agricultural than in forest systems.

## 4 DISCUSSION

Our study found over 670 macroinvertebrate morphospecies in the 18 sites from three Amazonian regions, including at least 70 new species of ecosystem engineers. The morphospecies richness observed at each site (min. 18 in agricultural, max. 91 in old forest) was within values reported for similar land uses in other Amazonian regions (Barros et al., 2006; Mathieu, 2004; Mathieu et al., 2005). We also found that although species richness was similar in ADE and REF soils, these two habitats harbor very different species pools, with few found in both habitats (Figure 3; Figure S2). This high turnover between sites and number of unique species appears to be a prevalent feature of Amazonian rainforest invertebrate communities (Maggia et al., 2021; Mathieu, 2004; Vasconcelos, 2006). Furthermore, although species rarefaction curves were still far from saturation with our current sampling effort, estimated richness showed similar trends, and showcased the wealth of species still to be discovered in both soils (Figure 4).

We believe that anthropic soils represent a major gap in the knowledge of Amazonian biodiversity. Soil animals have been poorly represented in taxonomic surveys in Amazonia (Constantino & Acioli, 2006; Franklin & Morais, 2006; James & Brown, 2006; Vasconcelos, 2006), and ADEs had not previously been sampled for soil macrofauna to this extent. Although ADEs occupy only a small fraction (0.1%–3%) of the Amazonian surface area (McMichael et al., 2014; Sombroek et al., 2003), they are scattered throughout the region (Clement et al., 2015; Kern et al., 2017), representing thousands of localized special habitats for species. The high β‐diversity values and species turnovers between different ADEs mean that each of these patches may be home to distinctive soil animal communities, including many new species, judging by the number of new ecosystem engineers found.

Soil provides chemical and physical support for vegetation and, as millennia of human activities created ADEs in the Amazon, patches with higher amounts of nutrients and organic resources were generated throughout a matrix of poorer soils (Kern et al., 2017; Macedo et al., 2019). The formation processes and human management of these soils result in distinct plant and microbial communities (Brossi et al., 2014; Clement et al., 2015; Levis et al., 2018; Taketani & Tsai, 2010), that are a result of disturbance, soil enrichment, and selection processes (both natural and human‐driven). Here we show that current soil animal abundance and diversity also reflect the impact of these ancient anthropogenic activities. The ADEs developed a different pool of species compared with REF soils. The former soils tend to favor more animals that recycle organic matter and flourish with higher pH and soil Ca, like earthworms and millipedes, while the latter favor termites, which are particularly sensitive to deforestation and changes in soil moisture and physical conditions (Dambros et al., 2013; de Souza & Brown, 1994; Duran‐Bautista, Muñoz Chilatra, et al., 2020; Eggleton et al., 1996). Similar microenvironmental characteristics of soil matrix and overlying vegetation probably have, and continue to influence soil fauna community composition in other anthropic soils in various regions of the world, such as in West Africa, Europe, and Central America (Macphail et al., 2017; Solomon et al., 2016; Wiedner et al., 2014). However, further elucidation of the pathways to changed community composition (and possibly species diversification) in ADEs and other anthropic soils would require expanding microbial and invertebrate biodiversity inventories.

The functional particularities observed in biotic communities of ADEs also mean that ecosystem functioning could be different in these soils, which could imply differences in their ecosystem services to humans, as observed in other human‐altered landscapes in Amazonia (Marichal et al., 2014; Rodríguez et al., 2021; Velásquez & Lavelle, 2019). Although relationships between the changes in macrofauna communities and soil aggregation, on the one hand, and ecosystem service delivery, on the other, have been mostly indirect (correlation rather than causation), it is well known that larger earthworm populations and improved soil structure owing mainly to fauna‐produced aggregates (as occurs in ADE) can alter soil hydraulic properties (Alegre et al., 1996; Hallaire et al., 2000), primary productivity (Brown et al., 1999; Pashanasi et al., 1996), litter decomposition, and nutrient cycling (Lavelle et al., 2006) as well as pedogenetic processes (Cunha et al., 2016; Macedo et al., 2017), and could help stabilize organic carbon in these soils (Cunha et al., 2016; Ponge et al., 2006). On the other hand, larger termite populations in REF soils could be contributing to ecosystem services as well (Duran‐Bautista, Armbrecht, et al., 2020), particularly in old forests, where fauna aggregates were also abundant. The links between soil fauna populations, land use, and ecosystems service delivery merit further attention, both in forested and agriculturally managed soils, particularly in ADEs.

As archeological sites, ADEs are protected by Brazilian law (Lei N^o^ 3.924 de 26 de Julho; Brasil, 1961), but throughout Amazonia they are actively sought out and intensively used for agricultural and horticultural purposes (Fraser et al., 2011; Junqueira et al., 2016; Kern et al., 2017). Intensive annual cropping and extensive livestock production represent a threat to soil macrofauna populations, both in REF and in ADE soils. Macroinvertebrate diversity in both soils decreased dramatically with increasing environmental disturbance (Figures 3 and 5), and negative impacts on some macroinvertebrate populations were higher in ADE than in REF soils. Modern human activity is often associated with negative environmental impacts in the Amazon (Decaëns et al., 2018; Franco et al., 2018), but on the other hand, the Pre‐Columbian historical human footprints associated with ADE formation processes and their long‐term traditional use appear to have “positive” effects on the Amazonian ecosystem (Balée, 2010). For instance, we found that old forests on ADEs were the most diverse LUS in terms of total soil macroinvertebrate morphospecies, and have also been shown to contain numerous useful tree and palm species (Levis et al., 2017, 2018).

Soil invertebrates are known to display high endemism (Lavelle & Lapied, 2003), and hence high β‐diversity values, mainly due to their low dispersal ability (Wu et al., 2011). Still, the high turnover rates between communities of ADE and REF soils suggest that ADEs may represent refuges for large numbers of specialist species that have been overlooked in previous work in the region (Barros et al., 2006; Constantino & Acioli, 2006; Franco et al., 2018; Vasconcelos, 2006), which has not targeted ADEs. This persistent anthropogenic footprint promotes biodiversity (Balée, 2010; Heckenberger et al., 2007) and modifies its distribution patterns in the Amazonian basin, showing that indigenous and traditional human populations and their activities are integral parts of the biome. This footprint is a prevailing driver in our study and, as such, should be integrated into future ecological research in Amazonia. Finally, considering their distinctive belowground communities, and the negative effect of modern land‐use intensification on their diversity and potential contributions to ecosystem service provisioning (Barros et al., 2006; Decaëns et al., 2018; Franco et al., 2018; Marichal et al., 2014), ADEs deserve special attention and management, to discover and protect their biological resources and promote more sustainable uses of Amazonian soils (Glaser, 2007).

## CONFLICT OF INTEREST

The authors declare no conflict of interests.

## Supporting information

Supplementary Material

## Data Availability

Demetrio, Wilian et al. (2020), A “Dirty” Footprint: Biodiversity in Amazonian Anthropogenic Soils, Dryad, Dataset https://doi.org/10.5061/dryad.3tx95x6cc (in review process).
