# Supplementary material for: A “Dirty” Footprint: Macroinvertebrate diversity in Amazonian Anthropic Soils
Source: Glob Chang Biol. 2021 Jul 10;27(19):4575–91. doi: 10.1111/gcb.15752 (PMC9292437; doi:10.1111/gcb.15752)
Supplement: Supplementary file 1 — Supplementary Material [file GCB-27-4575-s001.docx]

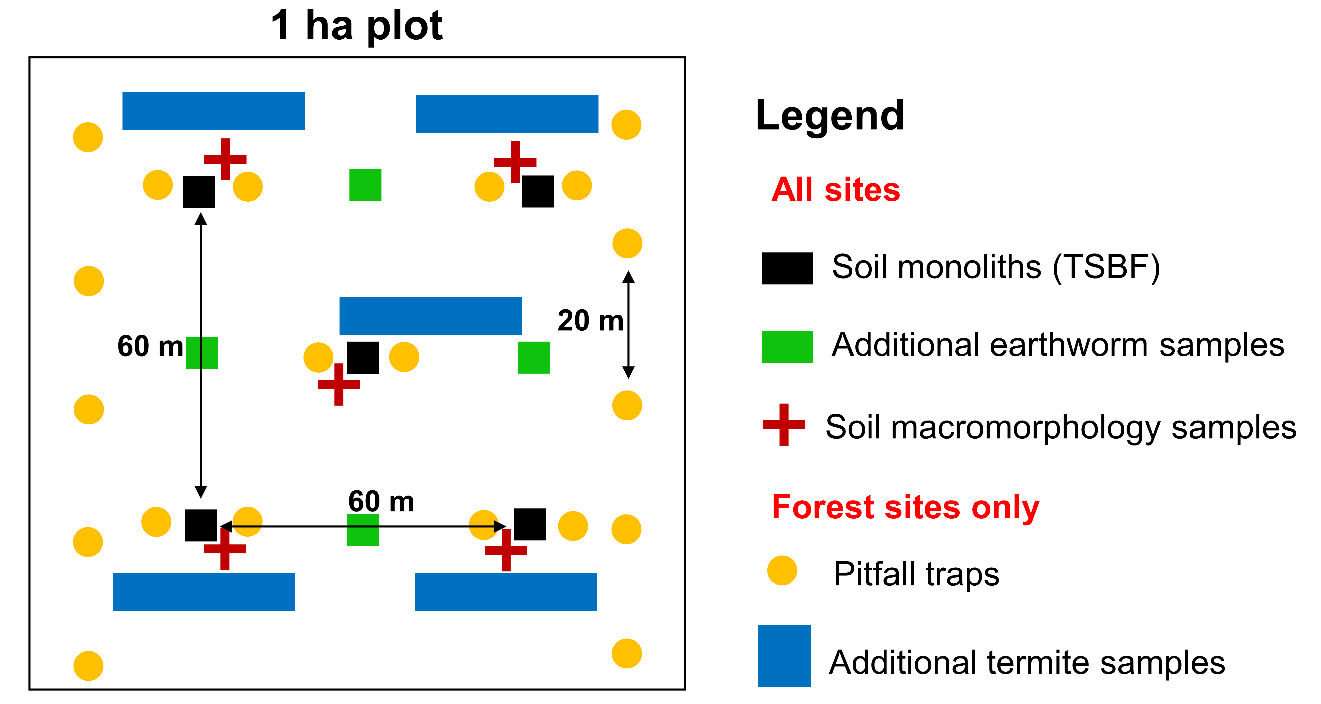


**Supplementary Figure 1.** Scheme used for soil and fauna sampling for each plot in each land use system. Distribution of the different samples in the 1 ha plot at all sites, showing the types of samples taken: monoliths for soil fertility and total soil macrofauna, soil macromorphology samples and additional samples for earthworms, and forest-only samples for termites (2 x 5 m plots) and ants (pitfall traps).





**Supplementary Figure 2.** Macroinvertebrate morphospecies richness collected in old secondary forests (O), young regeneration forests (Y) and agricultural systems (A) in reference (REF) and Amazonian Dark Earth (ADE) soils in three sampling regions (Iranduba, Belterra, Porto Velho). Unique species (singletons) are shown on the upper, unfilled part of each bar. The number of species shared by a soil category pair (ADE and REF) is shown above each pair of bars.


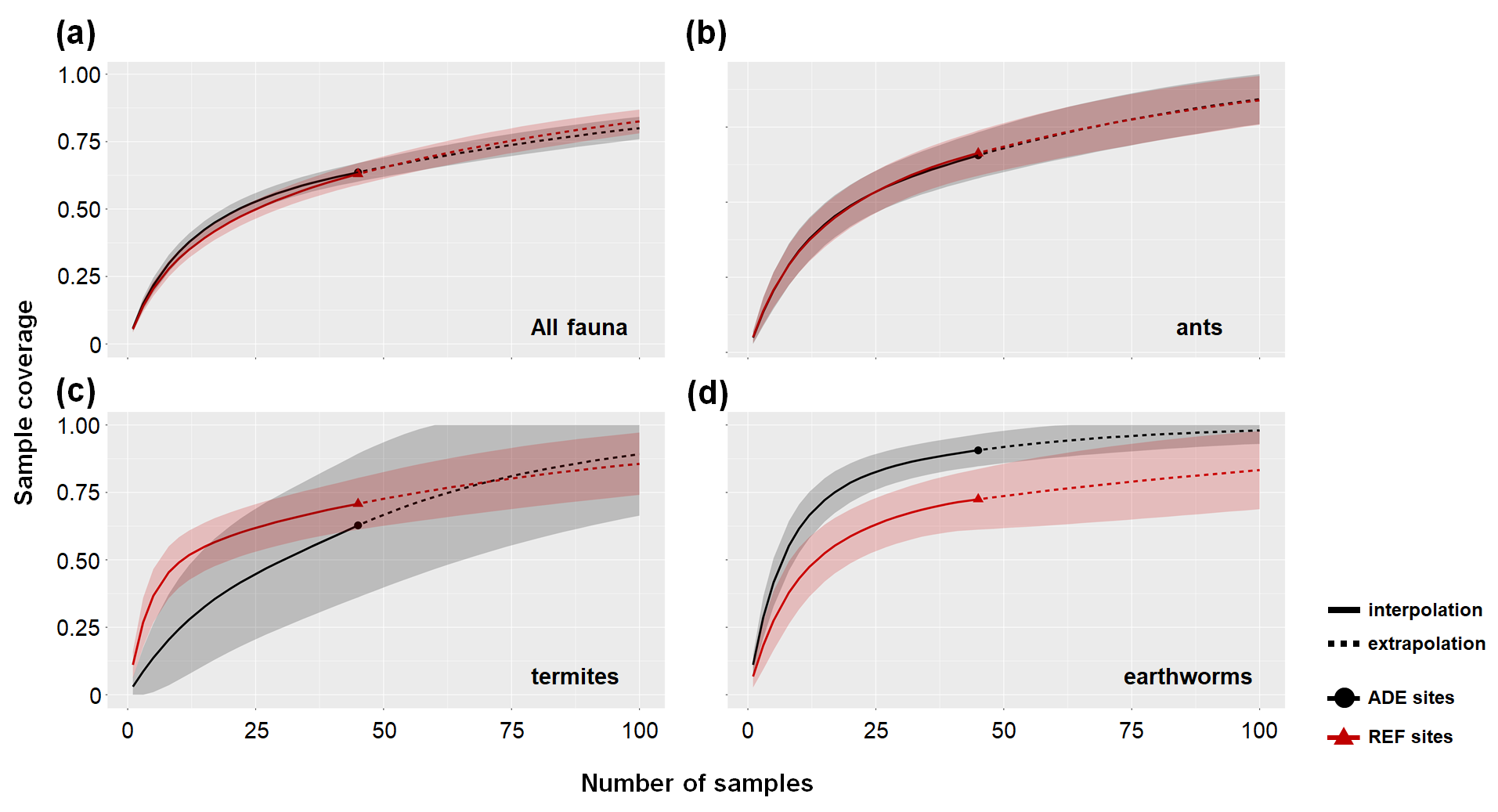


**Supplementary Figure 3.** Sampling effort coverage, showing diversity collected depending on sampling intensity (number of samples) in ADEs and REF soils for: (**a**) All soil macroinvertebrates, (**b**) ants, (**c**) termites and (**d**) earthworms. Data correspond to invertebrates collected using soil monoliths, over all sites and land use systems. Dark grey and red areas represent 95% confidence intervals. REF: Reference soil, ADE: Amazonian Dark Earth soil.

**Supplementary Table 1.** Soil analyses from the topsoil layers (0-30 cm depth) in reference (REF) and Amazonian Dark Earth (ADE) soils under each of the land-use systems (O: old secondary forests, Y: young regeneration forests, A: agricultural systems). Values represent means ± standard error from the three study regions. Upper case letters compare soil categories (ADE vs. REF), within each land-use system, while lower-case letters compare land use systems within the same soil type (ADE or REF). Different letters mean significant differences resulting from GLM. ns = non-significant.

| Soil type | Land use | K | CEC | Base saturation |  | Total N |  | Sand | Silt | Clay | Texture class (FAO) |
| --- | --- | --- | --- | --- | --- | --- | --- | --- | --- | --- | --- |
|  |  |  | | - - - - - - - - - - - - - - - - - - % - - - - - - - - - - - - - - - - - - | | | | | | |  |
| REF | O | 0.06±0.01Bb | 3.16±0.22Ba | 7.6±2.1Bb |  | 0.22±0.01Bb |  | 23±7^ns^ | 15±2Ab | 62±5Aa | Clay |
|  | Y | 0.05±0.0Bb | 2.89±0.09Ba | 1.2±0.1Bb |  | 0.27±0.03Ba |  | 26±4^ns^ | 22±2Aa | 52±2Ab | Clay |
|  | A | 0.09±0.01Ba | 3.16±0.34Ba | 19.5±5.7Ba |  | 0.21±0.01Bb |  | 21±4^ns^ | 19±9Aa | 60±3Aa | Clay |
| ADE | O | 0.07±0.01Ab | 8.76±0.78Aa | 55.1±4.9Aa |  | 0.28±0.02Ab |  | 23±5^ns^ | 17±2Ab | 60±4Aa | Clay |
|  | Y | 0.10±0.01Ab | 8.85±1.0Aa | 49.2±4.6Aa |  | 0.36±0.03Aa |  | 27±2^ns^ | 23±2Aa | 50±2Ab | Clay |
|  | A | 0.12±0.02Aa | 6.95±0.69Aa | 50.3±4.8Aa |  | 0.25±0.01Ab |  | 19±3^ns^ | 22±1Aa | 59±2Aa | Clay |

**Supplementary Table 2.** Number of singleton, doubleton, rare, and abundant species/morphospecies of all soil macroinvertebrate taxa and of selected macrofauna taxa collected in reference (REF) and Amazonian Dark Earth (ADE) soils (sum of all three regions and land use systems). Rare species represent taxa with fewer than 10 ind. over all samples. Non-rare and abundant species represent taxa with ≥10 ind. over all samples. Unique species were found in either ADE or REF; shared species were found in both soil categories. Species more abundant were considered to be those with quantities at least three times greater in ADE or REF soils, respectively. Locally distributed species were those found only in one region but in more than one sample site. Widely distributed species were those found in more than one region.

| Soil type | Taxon | Unique | Singletons |  | Doubletons | |  | Rare | |  | Non-rare or Abundant | | | | | | |
| --- | --- | --- | --- | --- | --- | --- | --- | --- | --- | --- | --- | --- | --- | --- | --- | --- | --- |
|  |  |  |  |  | Unique | Shared |  | Unique | Shared |  | Unique | Shared | More abundant in REF | Locally distributed in REF | More abundant in REF | Widely distributed (Total) | More abundant in REF |
| REF |  |  |  |  |  |  |  |  |  |  |  |  |  |  |  |  |  |
|  | Ants | 58 | 31 |  | 9 | 3 |  | 13 | 11 |  | 5 | 19 | 7 | 2 | 1 | 21 | 4 |
|  | Termites | 24 | 6 |  | 4 | - |  | 6 | 3 |  | 8 | 5 | 2 | 1 | - | 4 | 2 |
|  | Earthworms | 15 | 10 |  | 2 | - |  | 3 | 2 |  | - | 7 | - | 1 | - | 2 | - |
|  | Beetles | 35 | 28 |  | 3 | 4 |  | 4 | 4 |  | - | 4 | 1 | - | 1 | 3 | - |
|  | Millipedes | 16 | 5 |  | 3 | 4 |  | 8 | 4 |  | - | 5 | 2 | 1 | 2 | 1 | - |
|  | Total invertebrates | 287 | 178 |  | 37 | 14 |  | 56 | 44 |  | 16 | 53 | 14 | 7 | 5 | 41 | 8 |
| ADE |  |  |  |  |  |  |  |  |  |  |  |  | More abundant in ADE | Locally distributed in ADE | More abundant in ADE |  | More abundant in ADE |
|  | Ants | 62 | 27 |  | 10 | 3 |  | 16 | 11 |  | 9 | 19 | 7 | 1 | 2 | 21 | 7 |
|  | Termites | 5 | - |  | 2 | - |  | 2 | 3 |  | 1 | 5 | 1 | - | - | 4 | 1 |
|  | Earthworms | 15 | 2 |  | 3 | - |  | 6 | 2 |  | 4 | 7 | 5 | 4 | 5 | 2 | 2 |
|  | Beetles | 31 | 20 |  | 6 | 4 |  | 4 | 4 |  | 1 | 4 | 1 | - | - | 3 | 1 |
|  | Millipedes | 28 | 14 |  | 6 | 4 |  | 6 | 4 |  | 2 | 5 | 1 | - | 1 | 1 | - |
|  | Total invertebrates | 275 | 158 |  | 44 | 14 |  | 52 | 44 |  | 21 | 53 | 16 | 6 | 8 | 41 | 13 |

**Supplementary Table 3.** Mean biomass (fresh mass in g m^-2^± standard deviation) of the main soil macroinvertebrate taxa collected in each of the land-use systems (O: old secondary forests, Y: young regeneration forests, A: agricultural systems) studied in reference (REF) and Amazonian Dark Earth (ADE) soils. Different lower-case letters indicate significant differences between land-use systems within the same soil type, while different upper-case letters indicate significant differences between soil categories within each land-use system. Significance determined using GLMs or Kruskal-Wallis (KW) non-parametric tests.

| **GROUPS** | **REF** | | |  | **ADE** | | |
| --- | --- | --- | --- | --- | --- | --- | --- |
|  | **O** | **Y** | **A** |  | **O** | **Y** | **A** |
| **Earthworms¹** | 19.34±4.48Aa | 10.30±3.46Bab | 3.58±1.09Bb |  | 14.12±3.15Aa | 27.20±5.11Aa | 18.07±7.99Aa |
| **Termites¹** | 3.87±2.58Aa | 2.58±0.99Aa | 2.11±0.96Aa |  | 0.17±0.14Ba | 0.34±0.18Ba | 0.00±0.0Bb |
| **Ants¹** | 0.68±0.21Aa | 0.41±0.11Aab | 0.14±0.04Ab |  | 0.76±0.26Aa | 0.54±0.22Aa | 0.47±0.19Aa |
| **Ecosystem engineers¹** | 23.89±5.66Aa | 13.28±3.41Bab | 5.83±1.41Ab |  | 14.32±3.08Aa | 28.22±5.03Aa | 18.54±7.96Aa |
| **Beetles¹** | 1.73±1.07Ba | 2.60±1.18Aa | 0.58±0.18Aa |  | 3.11±0.96Aa | 0.58±0.20Ab | 0.21±0.11Ac |
| **Millipedes²** | 1.02±0.50Aa | 0.24±0.07Ba | 0.75±0.29Aa |  | 0.63±0.21Aa | 3.50±1.54Aa | 0.24±0.10Ab |
| **Centipedes²** | 0.49±0.23Aa | 0.29±0.09Aa | 0.03±0.01Ab |  | 0.27±0.05Aa | 0.31±0.11Ab | 0.11±0.06Ab |
| **Others²*** | 6.47±2.29Aa | 0.34±0.10Bb | 0.94±0.37Ab |  | 3.82±0.78Aa | 1.23±0.34Ab | 0.61±0.20Ab |
| **Total²** | 33.59±6.97Aa | 16.75±3.69Bab | 8.13±1.35Ab |  | 22.15±3.65Aa | 33.84±5.44Aa | 19.72±7.82Aa |

¹GLM; ²KW; *This group includes Orthoptera, Diptera (larvae), Dermaptera, Hemiptera, Isopoda, Blattaria, Gastropoda, Lepidoptera (larvae), Telyphonida, Solifugae, Opiliones, Scorpionida, Thysanoptera, Geoplanidae, Neuroptera (larvae), Hirudinea, Diplura, Vespidae and Embioptera
